# A multi-omic survey of black cottonwood tissues highlights coordinated transcriptomic and metabolomic mechanisms for plant adaptation to phosphorus deficiency

**DOI:** 10.3389/fpls.2024.1324608

**Published:** 2024-04-05

**Authors:** Emel Kangi, Edward R. Brzostek, Robert J. Bills, Stephen J. Callister, Erika M. Zink, Young-Mo Kim, Peter E. Larsen, Jonathan R. Cumming

**Affiliations:** ^1^ Department of Biology, West Virginia University, Morgantown, WV, United States; ^2^ Biology Department, Willamette University, Salem, OR, United States; ^3^ Biological Sciences Division, Pacific Northwest National Laboratory, Richland, WA, United States; ^4^ Loyola Genomics Facility, Loyola University Chicago, Maywood, IL, United States; ^5^ Department of Natural Sciences, University of Maryland Eastern Shore, Princess Anne, MD, United States

**Keywords:** phosphorus deficiency, cottonwood transcriptome, cottonwood metabolome, carbon metabolism, abiotic stress, populus trichocarpa

## Abstract

**Introduction:**

Phosphorus (P) deficiency in plants creates a variety of metabolic perturbations that decrease photosynthesis and growth. Phosphorus deficiency is especially challenging for the production of bioenergy feedstock plantation species, such as poplars (*Populus* spp.), where fertilization may not be practically or economically feasible. While the phenotypic effects of P deficiency are well known, the molecular mechanisms underlying whole-plant and tissue-specific responses to P deficiency, and in particular the responses of commercially valuable hardwoods, are less studied.

**Methods:**

We used a multi-tissue and multi-omics approach using transcriptomic, proteomic, and metabolomic analyses of the leaves and roots of black cottonwood (*Populus trichocarpa*) seedlings grown under P-deficient (5 µM P) and replete (100 µM P) conditions to assess this knowledge gap and to identify potential gene targets for selection for P efficiency.

**Results:**

In comparison to seedlings grown at 100 µM P, P-deficient seedlings exhibited reduced dry biomass, altered chlorophyll fluorescence, and reduced tissue P concentrations. In line with these observations, growth, C metabolism, and photosynthesis pathways were downregulated in the transcriptome of the P-deficient plants. Additionally, we found evidence of strong lipid remodeling in the leaves. Metabolomic data showed that the roots of P-deficient plants had a greater relative abundance of phosphate ion, which may reflect extensive degradation of P-rich metabolites in plants exposed to long-term P-deficiency. With the notable exception of the KEGG pathway for Starch and Sucrose Metabolism (map00500), the responses of the transcriptome and the metabolome to P deficiency were consistent with one another. No significant changes in the proteome were detected in response to P deficiency.

**Discussion and conclusion:**

Collectively, our multi-omic and multi-tissue approach enabled the identification of important metabolic and regulatory pathways regulated across tissues at the molecular level that will be important avenues to further evaluate for P efficiency. These included stress-mediating systems associated with reactive oxygen species maintenance, lipid remodeling within tissues, and systems involved in P scavenging from the rhizosphere.

## 1 Introduction

Phosphorus is an essential plant macronutrient that limits net primary production in many managed ecosystems. P deficiency results from the low natural abundance of soil P, removal of plant P during harvest, and the dependence of its bioavailability on soil pH (Marschner, 1995; Steen, 1998; Hinsinger, 2001; Raghothama and Karthikeyan, 2005; Cordell and White, 2014). Under P deficiency, plants experience disruptions in membrane integrity and the synthesis of key metabolites that result in reduced photosynthesis (Jacob and Lawlor, 1992; Lan et al., 2012). To counteract this deficiency, at the plant level, plants may invest more energy in root growth, mycorrhiza formation, and the release of organic acids and enzymes to mobilize soil P (Lan et al., 2015). At the ecosystem level, agricultural production adds roughly 16.5 megatons of P to ensure economically viable yields (Cordell and White, 2014). However, global rock phosphate reserves are being steadily depleted for fertilizer use (Steen, 1998). As such, there is a critical need to identify trait selection approaches to mediate plant P stress or select for beneficial plant traits to reduce fertilizer use. One pathway to establish the foundation for improving the ability of plants to overcome P deficiency lies in connecting the molecular underpinnings and the phenotypic response of plants to P deficiency.

Mitigating P deficiency is particularly important in ensuring sustainable bioenergy production to reduce fossil fuel emissions. Similar to traditional agriculture, there is a clear need to generate feedstocks that can be easily converted into biofuels with limited fertilizer inputs. *Populus trichocarpa* (herein black cottonwood) has emerged as a woody bioenergy feedstock because it is fast growing and can grow across a wide range of environmental conditions (Happs et al., 2020). In addition, black cottonwood has a fully sequenced genome and a large variety of genetic tools that can facilitate feedstock improvement (Tuskan et al., 2006; Douglas, 2017). Given efforts to produce bioenergy on marginal lands to reduce the displacement of food production, identifying the pathways by which black cottonwood responds to P deficiency at the phenotypic and molecular levels is essential to its use as bioenergy feedstock.

There is a rich body of research on the phenotypic response of plants to P deficiency. However, there remain key unknowns at the molecular level for the response of black cottonwood to P deficiency. Previous -omics research (defined as genomics, transcriptomics, proteomics, or metabolomics analyses) has identified key genetic, expression, and metabolic changes and pathways that shape plant responses to P deficiency and starvation (comprehensive reviews by Raghothama and Karthikeyan, 2005; Rouached et al., 2010; Plaxton and Tran, 2011; Lan et al., 2015). Many -omics studies have evaluated the responses of different -omes (defined as genomes, transcriptomes, proteomes, or metabolomes) to environmental stress. Much of this research, however, has been narrow in focus with studies limited to a specific plant tissue or a single -ome, e.g., only roots (Hernandez et al., 2007; Calderon-Vazquez et al., 2008; Li et al., 2008; Lan et al., 2012; Wang et al., 2021), only leaves (Müller et al., 2007; Zhang et al., 2014a; Muneer and Jeong, 2015), only transcriptome (Wu et al., 2003; Misson et al., 2005; Woo et al., 2012; Secco et al., 2013), only proteome (Yao et al., 2011). While there have been multiple -omics approaches that have successfully identified cross -ome linkages, these studies have used plant models relatively far removed from *P. trichocarpa*, such as legumes, C4 grasses, or plants adapted to P-deficient conditions (Liu et al., 2022; Luo et al., 2022; Liu et al., 2023). Studies with black cottonwood have focused on narrow gene families. A major gene family which has been extensively studied in *P. trichocarpa* in relation to P stress is the phosphate Pht1 transporter gene family (Loth-Pereda et al., 2011). In other models, such as Arabidopsis (*Arabidopsis thaliana*) and rice (*Oryza sativa*), P-starvation induced purple acid phosphatases and the PHO1 gene families have also been extensively studied and characterized (Zhang et al., 2011; Wang et al., 2004). Many SPX domain genes have also been studied for their involvement in plant P starvation and homeostasis in other important plant models (Duan et al., 2008; Secco et al., 2012; Du et al., 2017). Thus, there remain two key questions for black cottonwood: Are the changes in the transcriptomic, proteomic, and metabolomic profiles of black cottonwoods in response to P deficiency linked? Do roots and leaves have coordinated or unique molecular responses to P deficiency?

While evidence for a coordinated -omics response by black cottonwood to P deficiency is lacking, results from other model systems suggest that -omics responses to P deficiency fall on a spectrum from coordinated to disconnected. There are few P studies showing a concordant response of different -omes. A coordinated response of the transcriptome and the metabolome was found in P-deficient Arabidopsis *Arabidopsis thaliana* (Morcuende et al., 2007). Similarly, in P-deficient rice roots, the proteomic responses (Fukuda et al. (2007) mirrored transcriptomic changes under P deficiency (Wasaki et al. (2003). A coordinated response involving phenylpropanoid and flavonoid biosynthesis was detected in a multi-omics (lipidomics and transcriptomics) study of P-deficient *Pennisetum purpureum* (Luo et al., 2022). Finally, a degradation of phospholipids was detected in both the transcriptome and the lipidome of P-deficient *Cajanus cajan* (Liu et al., 2022). However, most studies in both Arabidopsis and other species have found that gene regulation, protein abundance, and metabolite abundance could be somewhat discordant (Li et al., 2008; Tran and Plaxton, 2008; Chen et al., 2011; Plaxton and Tran, 2011; Lan et al., 2012; Alexova and Millar, 2013; Lan et al., 2015). Therefore, the responses of different black cottonwood -omes to P deficiency may be similarly discordant, with each step dynamically controlled by both pre- and post-transcriptional regulation.

Similarly, the responses of different plant tissues to P deficiency may also be more complex, as some of them are likely whole-plant responses aimed at providing general abiotic stress protection, while others are tissue-specific and linked to the specialized functions of each tissue type. In response to P deficiency, most tissues would likely modify their membrane lipid composition, induce antioxidant defenses, and replace P-rich compounds with non-P containing substitutes (Dormann and Benning, 2002; Morcuende et al., 2007; Lei et al., 2011; Zhang et al., 2014b; Hernandez and Munne-Bosch, 2015). However, as different plant tissues have distinct functions, tissue-specific P deficiency responses are also likely. For example, leaves may invest in anthocyanin production to shed light energy that is not being harvested by photosynthesis (Hoch et al., 2001; Lei et al., 2011). By contrast, roots may change their architecture and morphology to improve P acquisition in shallow soil layers (Lynch, 2011). As such, identifying key pathways uniquely regulated in different tissues in response to P deficiency is critical for understanding the role of P in these different plant tissues and developing means of improving bioenergy feedstock production in P-deficient conditions.

Given these unknowns in the coordinated or disconnected responses of tissues and -omes to P deficiency in black cottonwood, the objectives of our study were to (1) link phenotypic responses in the leaves and roots of black cottonwoods undergoing P deficiency to the responses of the transcriptome, metabolome, and proteome and (2) identify harmonious and conflicting responses across -omes and tissues. In order to address these objectives, we grew seedlings of *P. trichocarpa*, a common hardwood model and bioenergy feedstock species, with deficient or replete P under controlled greenhouse conditions. We then quantified the phenotypic, transcriptomic, proteomic, and metabolomic responses of plants under these divergent conditions.

## 2 Materials and methods

### 2.1 Plant production and treatments

*Populus trichocarpa* Torr. & A. Gray ex Hook. seeds were obtained from the Canadian Natural Resources, National Tree Seed Center, P.O Box 4000, Fredericton NB, Canada. Seeds (lot 20037010.0) were collected in 2003 from a wild stand in Kamloops, British Columbia (50.6667˚N 120.2667˚W, elevation 345 m) and included seed from approximately 30 individual trees. Seeds were soaked in sterile water overnight before being transferred to 4 cm diameter × 18 cm deep pots (Conetainers™, Stuewe and Sons, Corvallis, OR, USA) containing sterilized acid-washed sand (coarse:fine sand ratio of 2:1) with nylon mesh covering the drainage holes. Germinated seeds were kept moist by watering 3 times daily to field capacity with 0.25 strength Johnson’s solution (Johnson et al., 1957). Following 7 days of germination, seedlings were thinned to leave single plants of similar size in each pot. Following thinning, seedlings were watered 3-times daily to field capacity (40 mL per pot) using modified Johnson’s solution with either 5 μM (P-deficient) or 100 μM (P-replete) of potassium phosphate (KH_2_PO_4_) with a pH of 5.6. We selected these levels based on previous studies of P-deficiency and P-stress responses in *Populus tremuloides* exposed to P levels ranging from 1 to 100 μM P (Desai et al., 2014; Shinde et al., 2017). Black cottonwoods were grown under greenhouse conditions with photoperiod augmented to 16 h with mixed metal halide lamps and a target temperature of 22/25˚C night/day. Relative humidity fluctuated with the weather. A total of 180 plants were grown for the study, with 90 plants grown in P-deficient and 90 plants grown in P-replete conditions. Plants were harvested at 71 days post planting. Plants were removed from pots, roots quickly rinsed free of sand, and plants separated into leaves, stems, and roots and processed as noted below. A total of 60 samples, 20 per -ome (5 plants per treatment × 2 P treatments × 2 tissues), were contributed to the transcriptome, proteome, and metabolome assessment pipelines.

### 2.2 Transcriptomics

Fresh tissues were immediately frozen in liquid nitrogen (LN_2_) and stored at –80˚C until further analysis. Within each P treatment, equal weights of leaf and root powder from 5 randomly selected black cottonwood seedlings were used for transcriptomics analyses. Tissue samples were crushed to a fine powder in LN_2_ using mortars and pestles, and RNA was extracted using Qiagen RNA Mini kits (Qiagen, Germantown, MD, USA) following manufacturer’s protocols. RNA was shipped to the U.S. Department of Energy’s Joint Genome Institute (JGI) for RNA expression profiling.

Stranded cDNA libraries were generated using the Illumina Truseq Stranded RNA LT kit. mRNA was purified from 100 ng of total RNA using magnetic beads containing poly-T oligos. mRNA was fragmented using divalent cations and high temperature. The fragmented RNA was reverse transcribed using random hexamers and SSII (Invitrogen™, Carlsbad, California, USA) followed by second strand synthesis. The fragmented cDNA was treated with end-pair, A-tailing, adapter ligation, and 10 cycles of PCR. The prepared libraries were quantified using KAPA Biosystems’ next-generation sequencing library qPCR kit and run on a Roche LightCycler 480 real-time PCR instrument. The quantified libraries were then prepared for sequencing on the Illumina HiSeq sequencing platform utilizing a TruSeq paired-end cluster kit, v4. Sequencing of the flowcell was performed on the Illumina HiSeq2500 sequencer using HiSeq TruSeq SBS sequencing kits, v4, following a 2 × 150 indexed run recipe.

Raw fastq file reads were filtered and trimmed using the JGI QC pipeline. Using BBDuk, raw reads were evaluated for artifact sequence by kmer matching (kmer=25), allowing one mismatch, and detected artifacts were trimmed from the 3’ end of the reads (Bushnell, 2014). RNA spike-in reads, PhiX reads, and reads containing any “N”s were removed. Quality trimming was performed using the phred trimming method set at Q6 (Ewing and Green, 1998). Finally, following trimming, reads under the length threshold were removed (minimum length 25 bases or 1/3 of the original read length, whichever was longer). Filtered reads from each library were aligned to the *P. trichocarpa* reference genome v3.0 using HISAT version 0.1.4-beta (Kim et al., 2015a). featureCounts was used to generate the raw gene counts using gff3 annotations (Liao et al., 2014). Only primary hits assigned to the reverse strand were included in the raw gene counts (-s 2 -p primary options). Raw gene counts were used to evaluate the level of correlation between biological replicates using Pearson’s correlation and determine which replicates would be used in the differential gene expression (DGE) analysis. *DESeq2* (version 1.10.0) was subsequently used to determine which genes were differentially expressed (DE) between pairs of conditions (Love et al., 2014). *DESeq2* has an internal quality control process, where genes with a low expression strength are filtered out before testing for differential expression. The parameters used to call a gene DE between conditions were p-adj < 0.05.

### 2.3 Proteomics

Proteins were extracted from plant tissues using a two-phase extraction protocol (Mitra et al., 2009; Nilsson et al., 2010; Abraham et al., 2013) modified here and described below. Briefly, approximately 2 g of root and leaves were ground using mortar and pestle under liquid nitrogen. Ground tissues were suspended in 30 mL protein extraction medium containing 0.33 M sucrose, 50 mM MOPS-KOH, pH 7.5, 5 mM EDTA, 0.2% (w/v) casein hydrolysate, and 10% (w/v) polyethylene glycol. Just prior to use, 0.6% (w/v) polyvinylpolypyrrolidone, 5 mM ascorbate and 5 mM DTT, and a protease inhibitor (Sigma P 9599) were added. The suspension was vortexed for 5 min, sonicated for 5 min in a cold-water bath, then centrifuged at 10,000 × g for 15 min. The supernatant was placed into a new tube and passed through a 0.45 μm filter then centrifuged at 100,000 × g for 2 hours. The resulting pellet was suspended in 10 mL of ice cold 0.1 M ammonium acetate in methanol and vigorously shaken, then placed at –20˚C for 2 hours or more. Samples were centrifuged again at 100,000 × g for 2 hours and supernatant discarded. The resulting pellet was dried using a gentle flow of nitrogen and then suspended in a protein solubilization solution (7 M urea, 2 M thiourea, 4% CHAPS) adding enough volume to cover the pellet plus an additional 500 µL. The suspension was allowed to rest overnight at 4˚C. After resting, the suspension was physically mixed using a pipettor and allowed to sit for 1 hour at 60˚C. After sitting, the suspension was vortexed briefly and centrifuged for 10 min at 5000 × g at 4˚C. The protein amount within the supernatant was determined using the BioRad Protein Assay Kit (Hercules, California, USA) using BSA as a standard and then digested with alkylation (chloroacetemide, 5 mM final concentration) using modified trypsin as previously described (Lin et al., 2017).

Tandem mass spectra (MS/MS) were generated using an LTQ-Orbitrap Velos MS (Thermo Scientific, Waltham, MA) coupled to a Waters NanoAcquity HPLC (Waters Corporation, Milford, MA). Eluting peptides were ionized using an ion funnel with nanoelectrospray source. HPLC conditions and instrument settings and have been previously published (Otwell et al., 2018). Raw data were de-isotoped using DeconMSn to identify parent ion masses based upon the THRASH algorithm (Horn et al., 2000). Peptide sequences from MS/MS were identified using the MSGF+ algorithm (Matzke et al., 2011) with a parameter file consisting of a 20 ppm MS tolerance window, inclusion of full and partial tryptic peptides, dynamic oxidized methionine (+15.994914), and static carbamidomethylation of cysteine residues (+57.021464). Searches were conducted against the *P. trichocarpa* genomic reference database (Poptr_1_1_JGI_2008-05-29; Populus_trichocarpa_210_v3_LegumeIP). MSGF+ searches included a decoy database approach for determining a false discovery rate (Elias and Gygi, 2007). Peptides filtered on a MSGF+ probability threshold of <1E–10 provided a peptide identification false discovery rate of <1%. Unique peptides were further filtered using a STAC score of greater than or equal to 0.5 (Stanley et al., 2011). Ion current arbitrary abundances were log_2_ transformed, normalized using their central tendency (Callister et al., 2006) and protein abundances estimated using the Rrollup algorithm available in the proteomics analysis software tool InfernoRDN (formerly DaNTE) (Polpitiya et al., 2008). Differential abundances of proteins were compared between low and high P using Welch’s t-test (Welch, 1947), with proteins having a p-value of < 0.01 retained.

### 2.4 Metabolomics

Metabolites were extracted from frozen and ground plant tissue samples by the protocol of MPLEx (modified Folch method) reported previously (Nakayasu et al., 2016). The extracted metabolites were completely dried under speed vacuum concentrator, then, chemically derivatized and analyzed by gas chromatography-mass spectrometry (GC-MS) as previously described (Kim et al., 2015b) by adding 20 µL of methoxyamine solution (30 mg/mL in pyridine) and incubated at 37˚C for 90 minutes to protect the carbonyl groups and reduce carbohydrate isoforms. Then 80 µL of N-methyl-N-(trimethylsilyl)-trifluoroacetamide with 1% trimethylchlorosilane were added to each sample to trimethylsilylate hydroxyl and amine groups for 30 mins.

Data collected by GC-MS were processed using the MetaboliteDetector software, version 2.5 beta (Hiller et al., 2009). Retention indices of detected metabolites were calculated based on analysis of the fatty acid methyl esters mixture (C8–C28), followed by chromatographic alignment across all analyses after deconvolution. Metabolites were initially identified by matching experimental spectra to a Pacific Northwest National Laboratory augmented version of the Agilent Fiehn Metabolomics Library containing spectra and validated retention indices for over 1000 metabolites (Kind et al., 2009), and additionally cross-checked by matching with NIST20 GC-MS Spectral Library and Wiley 11^th^ edition of GC-MS databases. All metabolite identifications were manually validated to minimize deconvolution and identification errors during the automated data processing.

### 2.5 Phenomics and biochemical analysis (tissue P)

Prior to harvest, chlorophyll fluorescence was assessed on all plants (N = 180) using an Opti-Sciences OS5p+ chlorophyll fluorescence meter (Opti-Sciences Inc, Hudson, NH, USA) and *F_v_/F_m_* was used as an indicator of photosynthetic function. For biomass measurements, 20 randomly chosen plants from each treatment group were harvested. Roots, shoots, and leaves were oven-dried at 60˚C for 72 h prior to being weighed. The dry weights of the roots and the aboveground components (both separately and together) were used to determine the root-to-shoot, leaf-to-shoot, and belowground-to-aboveground ratio of each plant.

For the determination of total total phosphorus concentrations in roots and leaves, samples (16 high P plants, 5 root and 3 leaf samples of low P plants) were dried, ground, and digested with nitric acid (HNO_3_) and hydrogen peroxide (H_2_O_2_) using a block digester (AD-4020, Westco Scientific Instruments, Brookfield, CT, USA). Digested samples were diluted with water, and vacuum filtered. The filtrate was further diluted to contain 1–5 ppm P using 3% HNO_3_. The total P concentrations of the leaf and root digests were measured using inductively coupled plasma optical emission spectrometry (ICP-OES, Agilent, Santa Clara, CA, USA).

### 2.6 Data analysis

Statistical tests on phenomics and biochemical data were performed using SAS JMP Pro 17 software (SAS Institute, Cary, NC, USA). Normality of the residuals was assessed using the Shapiro-Wilk test prior to further analysis. After normalization (when necessary), the effect of P treatment and tissue type on dry biomass, chlorophyll fluorescence, P concentration, and root-to-shoot ratio of dry biomass (P effect only) were evaluated using two-way analysis of variance (ANOVA).

The transcriptomics dataset was normalized through the internal normalization of DESeq2 (Love et al., 2014). For proteomics, redundant peptides within the false discovery rate filtered peptide results were combined by summing ion-current derived arbitrary abundances, then log_2_ transformed. Genes and enzymes were annotated using GO IDs and KEGG pathways. Gene annotations were taken from *P. trichocarpa* annotation v3.0 by Joint Genome Institute, as well as annotations from Gene Ontology (GO) and KEGG reaction and pathway databases. Metabolomics data were normalized by subtracting the mean for each metabolite; samples with fewer than 100 metabolites were excluded. Metabolites were annotated using KEGG pathways only. For the metabolomics dataset, we used a two-tailed t-test with a Benjamini-Hochberg correction to calculate significant differential expression (SDE). The same method was used to calculate SDE for the proteomics data.

Enriched GO and KEGG annotations in SDE genes and proteins were identified using hypergeometric distribution and calculated relative to the distribution of annotations present in the whole *P. trichocarpa* genome. The p-values obtained here indicate whether or not SDE’s annotation was SDE in each tissue separately (p_root_, p_leaf_), or in both tissues at the same time (p_both_). Binomial distribution was used to calculate directionality (i.e., up- or down-regulation) for the enrichment of GO and KEGG annotations in each tissue (p_posroot_, p_posleaf_). We selected a cutoff point of > 0.95 for upregulation and < 0.05 for downregulation. We also calculated the p-values of each annotation being enriched in the same direction (both up or both down) in both the roots and the leaves (p_same_). An α=0.05 was used for both p_both_ and p_same_, where p-values < 0.05 were considered to be significant.

Part of the statistical analysis for the proteomics and the metabolomics datasets was performed using R version 4.3.1 (2023-06-16) (R Core Team, 2023). To show the differences between P-deficient and control leaves and roots in the proteome and the metabolome, non-metric multidimensional scaling (NMDS) ordination plots were put together using R package ‘vegan’ using the Bray-Curtis dissimilarity index (Oksanen et al., 2022). Vectors shown on the ordination plots were selected based on proteins and metabolites with the strongest correlation (R^2^ > 0.8 cutoff for proteomics and selected groups for metabolomics) to be annotated (**Supplementary Materials**). For the metabolome, the abundance levels of metabolites selected as vectors were compared between the P-deficient and control tissues using a student’s t-test in SAS JMP Pro 17 software (SAS Institute, Cary, NC, USA). Also using the R package ‘vegan’ we ran a permutational multivariate analysis of variance (PERMANOVA, 999 permutations) to quantify the separation of the P treatment groups for proteomics and metabolomics (Oksanen et al., 2022). Results of the PERMANOVA for proteomics and metabolomics, as well as comparisons between normalized metabolite abundances in P-deficient and control plants using a Benjamini-Hochberg correction can be found in the **Supplementary Materials** in folders for the respective -omics dataset.

## 3 Results

### 3.1 Phenomics: P deficiency reduced black cottonwood growth and PSII activity

**Figure 1:**
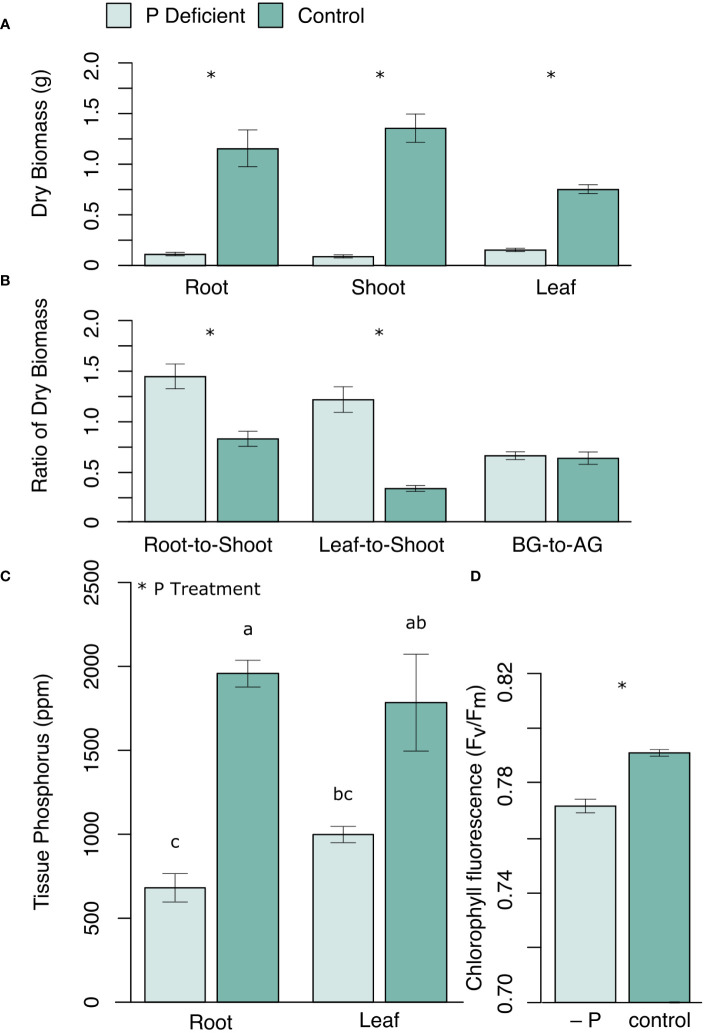
**(A)** Mean dry biomass (g) of roots, shoots and leaves, and **(B)** the ratios of dry biomass (root-to-shoot, leaf-to-shoot, and belowground dry biomass to aboveground dry biomass (BG-to-AG)), as well as **(C)** mean tissue P concentration (ppm) in roots and leaves, and **(D)** leaf chlorophyll fluorescence *(F_v_/F_m_*) in P-deficient (light green) and control (green) plants. Error bars represent means ± standard error. * *p <*0.05. For **(C)** bars assigned the same letter are not significantly different from each other after Tukey HSD (*p* < 0.05).

Phosphorus (P) deficiency significantly reduced black cottonwood growth in all three tissue types (roots, shoots, and leaves) (**Figure 1A**). The root-to-shoot ratio and the leaf-to-shoot ratio of dry biomass were both greater in P-deficient plants than in P-replete plants (**Figure 1B**). However, the ratio of the belowground dry biomass to the aboveground dry biomass (leaf + shoots) was not significantly different between the treatment groups (**Figure 1B**), reflecting the disproportional reduction in woody biomass under P deficiency. Chlorophyll *F_v_/F_m_* was also lower in P-deficient plants (**Figure 1D**). Phosphate deficient plants had significantly lower tissue P levels than control plants (**Figure 1C**; p_root_ < 0.0001; p_leaf_ = 0.0003).

### 3.2 Transcriptomics: leaves had a greater number of differentially expressed genes than roots in response to P deficiency

The RNA-seq analysis produced 74.4–100.1 million raw reads for the root replicates, and 76.1–163.8 million raw reads for the leaf replicates (**Supplementary Table 2**). The average read length was 151 bp. A total of 255.6 Gb of clean bases remained after filtering for quality. Reads were correctly mapped to the *P. trichocarpa* reference genome with a mapping ratio of 94.5–98.6 (**Supplementary Table 2**).

**Figure 2:**
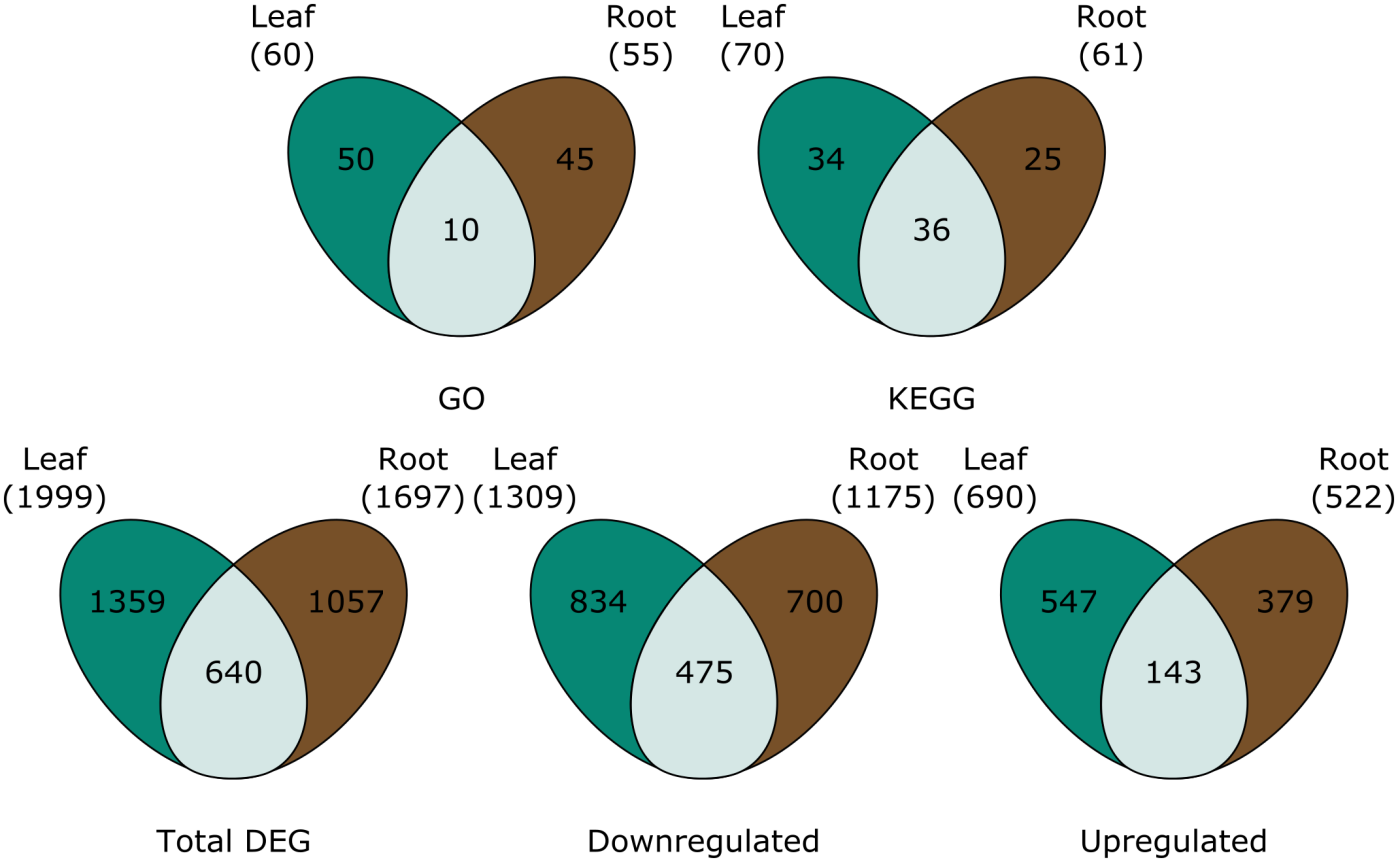
Venn diagrams are showing (top row) the number of GO terms and KEGG pathways assigned to all significantly differentially expressed genes, and (bottom row) the total number of differentially expressed genes (total DEG), downregulated genes, and upregulated genes in the leaves (green), roots (brown) and both (light green). For GO and KEGG, pathways were only considered differentially expressed in both the leaves and the roots when tissue overlap was statistically significant.

A total of 1999 genes were differentially expressed in P-deficient leaves, 1359 of which were unique to the leaves (**Figure 2**). In the roots, 1697 genes, 1057 of which were tissue-specific, were differentially expressed in response to P deficiency (**Figure 2**). Among downregulated genes, a greater percentage of differentially expressed genes were shared between tissues (23.6%), than among upregulated genes (11.8%) (**Figure 2**).

The top six upregulated leaf genes with the greatest log_2_FC were genes encoding phosphorylase superfamily proteins (log_2_FC between 15 and 12) (**Supplementary Table 3**). Among the annotated differentially expressed genes in the leaves, the top ten genes with the greatest negative log_2_FC included 3 genes encoding ​​beta-glucosidases (one with log_2_FC = –12 and two with log_2_FC = –11) and three genes encoding berberine bridge enzymes (log_2_FC = –12, all) (**Supplementary Table 3**). Other downregulated genes in the leaves were annotated as allene oxide synthase (log_2_FC = –12), acyl-CoA N-acetyltransferase (NAT) superfamily protein (log_2_FC = –12), and FAD-binding berberine family protein (log_2_FC = –12) (**Supplementary Table 3**).

Upregulated root genes with the greatest log_2_FC included AMP-dependent synthetase and ligase family protein (log_2_FC = 10), aluminum-activated malate transporter family protein (log_2_FC = 9.2), 3-ketoacyl-CoA synthase 6 (log2FC = 8.6), SPX domain gene 3 (log_2_FC = 7.3), and purple acid phosphatase 17 (log_2_FC = 5.7) (**Supplementary Table 3**). Downregulated root genes with the greatest log_2_FC included cytochrome P450, family 71, subfamily B, polypeptide 34 (log_2_FC = –15), HXXXD-type acyl-transferase family protein (log_2_FC = –10), galactinol synthase 1 (log_2_FC = –10), and senescence-associated gene 29, which encodes SWEET15 (log_2_FC = –9.5) (**Supplementary Table 3**).

### 3.3 Transcriptomics: more GO terms related to growth were downregulated in leaves than roots in response to P deficiency

Although there was a high level of tissue specificity in GO terms assigned to significantly differentially expressed genes, genes in two GO terms were regulated similarly across leaves and roots (**Figure 2**). DNA-directed RNA polymerase activity was upregulated in both tissue types (**Table 1**). In contrast, cation transport was downregulated for both tissue types separately (**Table 1**).

**Table 1:** Significantly up/downregulated (red and blue, respectively) GO terms for the differentially expressed genes in the leaves (green), roots (brown) and both tissues (light green), together with the functional categories assigned to them are listed in the table below.

	**Leaf**		**Upregulated**		
	**Both**		**Downregulated**		
	**Root**				
	**Functional Category**		**GO ID**	**Go Annotation**	**SDE p-adj**
	Protection Unassigned		GO:0016671 GO:0050661	Oxidoreductase activity, acting on a sulfur group of donors, disulfide as acceptor NADP bindi	1e-2 3e-2
	Growth/Energy Signaling Unassigned		GO:0010215 GO:0033178 GO:0016049 GO:0008295 GO:0006812 GO:0031225 GO:0016790 GO:0003935	Cellulose microfibril organization Proton-transporting two-sector ATPase complex, catalytic domain Cell growth Spermidine biosynthetic process **Cation transport** Anchored component of membrane Thiolester hydrolase activity GTP cyclohydrolase II activity	8e-4 2e-2 3e-2 3e-2 2e-2 8e-4 2e-3 1e-2
	Unassigned		GO:0003899	DNA-directed 5’-3’ RNA polymerase activity	4e-3
	Signaling Epigenetic Changes Unassigned		GO:0045017 GO:0045017 GO:0005787 GO:0006465 GO:0035267 GO:0031491 GO:0006812 GO:0008483 GO:0006024 GO:0015012	Glycerolipid biosynthetic process Glycerolipid biosynthetic process Signal peptidase complex Signal peptide processing NuA4 histone acetyltransferase complex Nucleosome binding **Cation transport** Transaminase activity Glycosaminoglycan biosynthetic process Heparan sulfate proteoglycan biosynthetic process	5e-5 4e-3 5e-2 1e-2 3e-2 5e-2 2e-2 1e-2 1e-2

Pathways differentially expressed in both tissues with no significant overlap are shown in bold.

The p-values represent enrichment in the respective tissues, or (for both) enrichment in both tissues.

When observing each tissue type individually, the leaf tissues had three significantly upregulated GO terms in response to P deficiency (**Table 1**). Of the three upregulated leaf terms, two could not be classified, but the remaining term was related to tissue protection/reactive oxygen species (ROS) scavenging. No GO groups were significantly upregulated exclusively in roots. Among the significantly downregulated GO terms, leaf terms were mainly related to growth and energy and signaling (**Table 1**). On the other hand, significantly downregulated GO terms for root tissues were broadly classified as belonging to signaling and epigenetic modifications groups (**Table 1**).

It is important to note that not every GO term could be neatly classified, as many terms can have a very broad range of functions. Further, when evaluating genes or metabolites using GO or KEGG pathways, detected differences in significance may not be assigned directionality because some genes in a pathway may be upregulated while others downregulated. In these cases, we simply imply significance in expression/concentration.

### 3.4 Transcriptomics: leaves have more downregulated KEGG pathways than roots in response to P deficiency

Many of the KEGG pathways assigned to the significantly differentially expressed genes were present in both the leaves and the roots (**Figure 2**; **Tables 2**, **3**). Of the KEGG pathways that were unique to leaves or roots, more were downregulated in the leaves than in the roots (**Table 2**).

**Table 2:** Significantly up/downregulated (red and blue, respectively) KEGG pathways for the differentially expressed genes and the functional categories assigned to them are shown for the leaves (green) and the roots (brown).

	**Leaf**		**Upregulated**		
	**Root**		**Downregulated**		
**Functional Category**			**ID**	**Annotation**	**SDE p-adj**
	Amino Acids 2° Metabolites Hormones Enregy Protection		400 950 945 904 20 941	Phenylalanine, tyrosine & tryptophan biosynthesis Isoquinoline alkaloid biosynthesis Stilbenoid, diarylheptanoid & gingerol biosynthesis Diterpenoid biosynthesis Citrate cycle (TCA cycle) Flavonoid biosynthesis	2e-8 2e-4 8e-5 8e-5 3e-8 3e-6
	Energy Signaling Prot. Synthesis Hormones Unassigned		52 590 591 561 290 970 360 340 982 680 740 830	Galactose metabolism **Arachidonic acid metabolism** Linoleic acid metabolism Glycerolipid metabolism Valine, leucine & isoleucine biosynthesis Aminoacyl-tRNA biosynthesis Phenylalanine metabolism Histidine metabolism **Drug metabolism – cytochrome P450** Methane metabolism Riboflavin metabolism Retinol metabolism	9e-7 1e-2 1e-2 2e-5 2e-3 3e-4 5e-2 1e-3 2e-3 9e-3 4e-3 4e-2
	2˚ Metabolites Signaling Hormones		903 750 281 72 650 982	Limonene & pinene degradation Vitamin B6 metabolism Geraniol degradation Synthesis & degradation of ketone bodies Butanoate metabolism **Drug metabolism – cytochrome P450**	6e-3 2e-3 2e-2 6e-3 5e-4 2e-3
	Signaling Unassigned		590 564 603 670	**Arachidonic acid metabolism** Glycerophospholipid metabolism Glycosphingolipid biosynthesis – globo series One carbon pool by folate	3e-4 2e-4 4e-3 4e-7

Pathways differentially expressed in both tissues with no significant overlap are shown in bold.

The p-values represent enrichment in the respective tissues, or (for both) enrichment in both tissues.

**Table 3:** Significantly up/downregulated (red and blue, respectively) KEGG pathways for the differentially expressed genes and the functional categories assigned to them are shown for pathways that show significant overlap in both tissues (light green).

	**Both**		**Upregulated**		
			**Downregulated**		
			**Any Direction**		
**Functional Category**			**ID**	**Annotation**	**SDE p-adj**
	Signaling Prot. Degradation		600 310	Sphingolipid metabolism Lysine degradation	3e-2 4e-2
	Energy Signaling Prot. Metabolism Protection Hormones 2˚ Metabolites Unassigned		710 860 51 10 500 620 760 61 562 520 260 965 920 53 480 100 521 240 604 531 625 790 626	Carbon fixation in photosynthetic organisms Porphyrin and chlorophyll metabolism Fructose & mannose metabolism Glycolysis/Gluconeogenesis Starch & sucrose metabolism Pyruvate metabolism Nicotinate & nicotinamide metabolism Fatty acid biosynthesis Inositol phosphate metabolism Amino sugar & nucleotide sugar metabolism Glycine, serine & threonine metabolism Betalain biosynthesis Sulfur metabolism Ascorbate & aldarate metabolism Glutathione metabolism Steroid biosynthesis Streptomycin biosynthesis Pyrimidine metabolism Glycosphingolipid biosynthesis – ganglio series Glycosaminoglycan degradation Chloroalkane & chloroalkene degradation Folate biosynthesis Naphthalene degradation	1e-8 3e-4 8e-3 1e-6 4e-2 6e-3 1e-6 2e-4 9e-4 2e-5 2e-2 1e-4 2e-2 6e-3 5e-9 4e-5 4e-7 3e-4 4e-7 3e-2 2e-2 9e-14 2e-2
	Prot. Metabolism 2˚ Metabolites Hormones Signaling Unassigned		250 330 350 910 750 780 904 407 621 565 450	Alanine, aspartate & glutamate metabolism Arginine & proline metabolism Tyrosine metabolism N metabolism Vitamin B6 metabolism Biotin metabolism Diterpenoid biosynthesis Phosphatidylinositol signaling system Dioxin degradation Ether lipid metabolism Selenocompound metabolism	2e-7 2e-4 2e-7 3e-7 5e-2 3e-2 3e-9 8e-3 3e-6 4e-5 1e-2

The p-values represent enrichment in both tissues.

There was little overlap between the functional categories assigned to upregulated KEGG pathways in the leaves and in the roots. Of the significantly enriched KEGG pathways assigned to the leaf transcriptome of P-deficient plants, significantly upregulated pathways were involved in amino acid metabolism, secondary metabolites, phytohormones, energy metabolism, and protection/ROS scavenging (**Table 2**). In contrast, the KEGG pathways upregulated in the transcriptome of P-deficient roots were most frequently classified as belonging to secondary metabolite degradation, followed by signaling and phytohormones (**Table 2**). Sphingolipid Metabolism (map00600) and Lysine Degradation (map00310) had cross-tissue overlap, and these were both upregulated (**Table 3**). In addition, there were two pathways that were statistically significantly differentially expressed in both tissue types together while only being significantly upregulated in one tissue type. These pathways were Vitamin B6 Metabolism (map00750) and Diterpenoid Biosynthesis (map00904) (**Table 3**).

There was some overlap in the functional classifications assigned to downregulated pathways in the leaves and roots of black cottonwood. The pathways downregulated exclusively in the leaves were mainly related to protein synthesis, signaling, phytohormone metabolism, protection/ROS scavenging, and energy (**Table 2**). A few pathways could not be assigned a broad functional category, as these pathways could have a large variety of roles (**Table 2**). On the other hand, there were only four pathways downregulated exclusively in the roots, of which two could be assigned to signaling and two were not assigned to a functional category (**Table 2**). Arachidonic Acid Metabolism (map00590) was downregulated in both the leaves and the roots separately, but neither the enrichment nor the directionality significantly overlapped between the tissues (**Table 2**).

Many pathways were significantly differentially expressed in both leaf and root tissues (**Table 3**). However, the agreement between the tissues in terms of the directionality (i.e., both up-regulated or both down-regulated) of these pathways was not always statistically significant (**Table 3**). These pathways were assigned to energy, signaling, protein metabolism, protection/ROS scavenging, phytohormone metabolism and secondary metabolite synthesis (**Table 3**). Some of the pathways could not be properly classified (**Table 3**).

### 3.5 Metabolomics: significantly enriched KEGG pathways primarily supported transcript data

**Figure 3:**
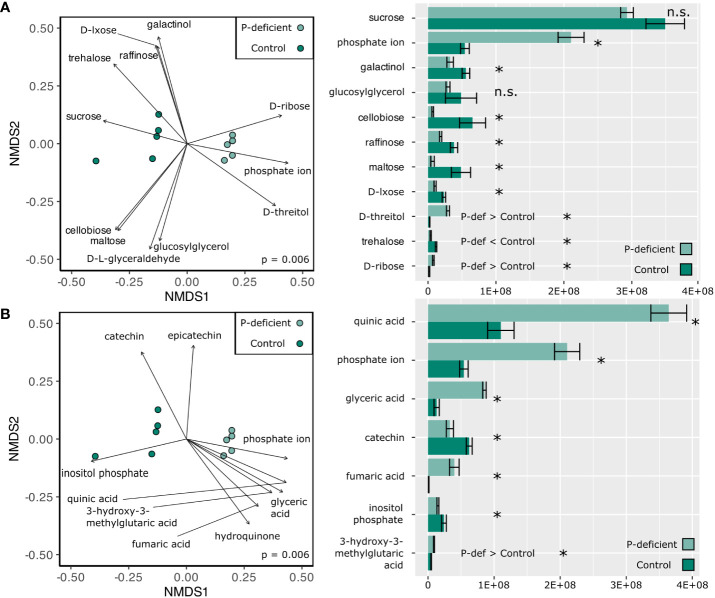
(Left) Non-metric multidimensional scaling (NMDS) based on the metabolite abundances of the P-deficient (light green) and the control (green) plants in the leaves. Vectors represent selected metabolites involved in **(A)** primary C metabolism and **(B)** secondary metabolism with a statistically significant relationship (p < 0.05) with the NMDS regression. Results of a PERMANOVA are indicated by the p-value on the NMDS plots. (Right) Mean abundance values of selected metabolites ± standard error. Asterisks (*) indicate that the abundance of a metabolite is significantly different between P-deficient (light green) and control (green) plants (* = p < 0.05, n.s. = not significantly different, student’s t-test). P-def < Control and P-def > Control indicate that the low-abundance metabolites were respectively lower or greater in the P-deficient leaves.

**Figure 4:**
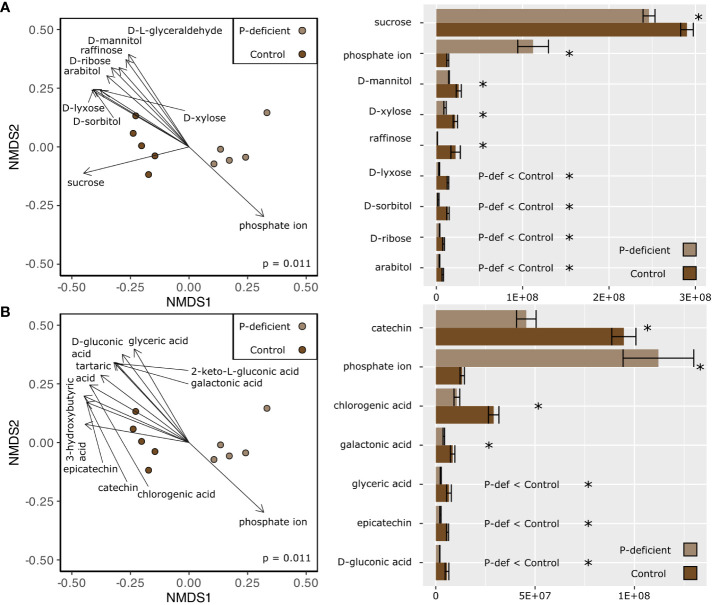
(Left) Non-metric multidimensional scaling (NMDS) based on the metabolite abundances of the P-deficient (light brown) and the control (brown) plants in the roots. Vectors represent selected metabolites involved in **(A)** primary C metabolism and **(B)** secondary metabolism with a statistically significant relationship (p < 0.05) with the NMDS regression. Results of a PERMANOVA are indicated by the p-value on the NMDS plots. (Right) Mean abundance values of selected metabolites ± standard error. Asterisks (*) indicate that the abundance of a metabolite is significantly different between P-deficient (light brown) and control (brown) plants (* = p < 0.05, student’s t-test). P-def < Control and P-def > Control indicate that the low-abundance metabolites were respectively lower or greater in the P-deficient roots.

Non-metric multidimensional scaling (NMDS) based on the expression in the metabolome showed differences between the P-deficient and the control plants in both the leaf and the root tissues (**Figures 3**, **4**; p_leaf_ = 0.006; p_root_ = 0.011, PERMANOVA). Selected metabolites involved in primary C metabolism and secondary metabolism were included as vectors on the NMDS plots (**Figures 3A**, **4A**; **Figures 3B**, **4B**). Control leaves had a greater abundance of most primary C metabolites, with the exception of D-threitol and D-ribose (**Figure 3A**). Of the secondary metabolites, P-deficient leaves had a greater abundance of quinic, glyceric, fumaric, and 3-hydroxy-3-methylglutaric acids (**Figure 3B**). P-limited roots had a lower abundance of all of the selected metabolites (**Figures 4A, B**).

Only leaf tissues had significantly upregulated KEGG pathways in the metabolomics dataset (**Table 4**). These pathways were annotated as Benzoate Degradation (map00362) and Starch and Sucrose Metabolism (map00500) (**Table 4**). The directionality of the other significantly differentially expressed KEGG pathways in the leaves was not statistically significant. The pathways differentially expressed in the leaves were broadly classified into functional categories for signaling changes, energy metabolism, and oxidative stress response (**Table 4**).

**Table 4:** Significantly differentially expressed KEGG pathways (upregulation shown in red) for the metabolites in the leaves (green), roots (brown) and both (light green).

	**Leaf**		**Upregulated**	
	**Both**		**Any Direction**	
	**Root**			
	**KEGG ID**		**KEGG Annotation**	**SDE p-adj**
	362 500		Benzoate degradation Starch & sucrose metabolism	2e-3 5e-5
	52 361 624 53		Galactose metabolism Chlorocyclohexane & chlorobenzene degradation Polycyclic aromatic hydrocarbon degradation Ascorbate & aldarate metabolism	8e-3 1e-2 9e-4 3e-2
	400		Phenylalanine, tyrosine & tryptophan biosynthesis	5e-4
	230 20 630 53		Purine metabolism Citrate cycle (TCA cycle) Glyoxylate & dicarboxylate metabolism Ascorbate & aldarate metabolism	3e-5 6e-5 1e-2 6e-2

The p-values represent enrichment in the respective tissues or (for both) enrichment in both tissues.

For roots, KEGG pathways under the broad functional categories of protection/ROS scavenging, detoxification, resource/P scavenging, and energy metabolism were significantly differentially expressed (**Table 4**). The pathway for Ascorbate and Aldarate Metabolism (map00053) was significantly differentially expressed in both the leaves and the roots (**Table 4**).

### 3.6 Proteomics: differences in GO term enrichments were not detected

637021 peptides, 99% of which had a false discovery rate of less than 0.05, were detected in all of the leaf samples. Of those, 4583 peptides identified with only 1 protein. Including contaminants, 693 unique proteins were detected in at least 1 of the leaf samples. Including contaminants, 240 unique proteins were detected in at least half of the leaf samples. After contaminants were removed, 234 unique proteins were detected in at least half of the leaf samples.

933734 peptides, 99% of which had a false discovery rate of less than 0.05, were detected in all of the root samples. Of those, 7614 peptides identified with only 1 protein. Including contaminants, 1072 unique proteins were detected in at least 1 of the root samples. Including contaminants, 416 unique proteins were detected in at least half of the root samples. After contaminants were removed, 410 unique proteins were detected in at least half of the root samples.

Non-metric multidimensional scaling (NMDS) based on the expression in the proteome showed differences between the P-deficient and the control plants in both the leaf and the root tissues (**Supplementary Figure 1**; p_root_ < 0.006; p_leaf_ = 0.01, PERMANOVA).

One protein encoded by the gene (Potri.017G037900.1) was likely a peroxidase responsible for oxidative stress response and was significantly enriched in the leaves (log_2_FC of 8.02, p = 0.02, **Supplementary Materials**). In the roots, a universal stress protein encoded by the gene (Potri.T120500.1) was significantly enriched (log_2_FC of 9.48, p = 0.01, **Supplementary Materials**).

However, we detected no statistically significantly enriched GO terms in our P-deficient plants (**Supplementary Materials**).

### 3.7 Inter-omic comparison shows overlapping and conflicting pathways

Several differentially expressed KEGG pathways were commonly detected across the tissues and -omes, but their directionality varied. For example, the KEGG pathway for Ascorbate and Aldarate Metabolism (map00053) was downregulated in the transcriptome and significantly differentially expressed in the metabolome (**Tables 3**, **4**). Similarly, the KEGG pathway for Phenylalanine, Tyrosine, and Tryptophan Biosynthesis (map00400) was upregulated in the leaf transcriptome, and significantly differentially expressed in the metabolome (**Tables 2**, **4**). On the other hand, the KEGG pathway for the Starch and Sucrose Metabolism (map00500) was downregulated in the black cottonwood transcriptome as a whole, but the same pathway was upregulated in the leaf metabolome (**Tables 3**, **4**).

## 4 Discussion

This work uncovers key molecular mechanisms underlying the coordinated responses of black cottonwood leaves and roots to P deficiency. We found that while both the aboveground and the belowground tissues limit growth and energy production in response to P deficiency, this suppression is greatest in the aboveground woody biomass (**Figure 1A**). In line with this finding, we observed that the stressed black cottonwoods allocated less biomass to the shoot tissues, relative to the root and the leaf tissues (**Figure 1B**). Additionally, changes in the leaf tissue transcriptome were more greatly regulated than root tissues, with a higher number of DEGs as well as a greater number of downregulated KEGG pathways in response to P deficiency than the root transcriptome (**Figure 2**; **Table 2**). Meanwhile, the roots exhibited standard P starvation responses, including the upregulation of genes encoding the aluminum activated malate transporter family protein, purple acid phosphatase 17, and SPX domain gene 3, all underpinning the physiological mechanisms of P scavenging from the soil (**Supplementary Table 3**) (Balzergue et al., 2017; Mora-Macias et al., 2017; Liu et al., 2018; Kavka et al., 2021). The leaf tissues, in particular, also displayed a significant decline in the efficiency of photosynthetic machinery, both in the phenome and the transcriptome (**Figure 1D**; **Table 4**, map00710). Coupled with the increases in both the root-to-shoot and the leaf-to-shoot ratios of dry biomass in P-deficient plants, this finding further supports the idea that P-deficient black cottonwoods are prioritizing resource acquisition at the expense of woody biomass production (**Figure 1B**). Although we did not find any significant changes to the proteome, 60% of the pathways that were altered in the metabolome of the P-deficient plants were also altered in the transcriptome. Collectively, these results demonstrate the tissue-specificity of black cottonwood P deficiency responses, highlighting the importance of comparing different plant tissues in future black cottonwood-environment studies. In addition, this work reveals several pathways that were regulated in both the transcriptome and the metabolome of P-deficient black cottonwoods, making them likely candidates for key regulators of black cottonwood P deficiency response.

P-deficient black cottonwood seedlings had lower aboveground and belowground biomass than P-replete seedlings, which paralleled the downregulation of GO terms and KEGG pathways for genes corresponding to growth and C metabolism in P-deficient black cottonwoods (**Figure 1**; **Tables 1**, **3**). There were multiple findings that supported this connection between lower observed growth and downregulation of pathways that control biomass production. First, we found that the GO term for Cell Growth (GO:0016049) was downregulated in the leaves of P-deficient plants (**Table 1**). Similarly, both the KEGG pathway for Starch and Sucrose Metabolism (map00500) as well as the pathway for Fructose and Mannose Metabolism (map00051) were downregulated in the transcriptome of both the leaves and the roots of P-deficient plants (**Table 3**). P-deficient plants alter their carbohydrate metabolism and accumulate starch and sugars in specific tissues in response to P deficiency (Ariovich and Cresswell, 1983; Fredeen et al., 1989; Cakmak et al., 1994). Under mild P deficiency, this accumulation of sugars can lead to the feedback inhibition of photosynthesis, while under severe P deficiency, reductions in photosynthesis can arrest starch accumulation altogether (Plaut et al., 1987; De Groot et al., 2001; Wu et al., 2003; Hermans et al., 2006). Furthermore, in the current study, KEGG pathway analysis of the transcriptome showed that, in P-deficient plants, the pathways responsible for Glycolysis/Gluconeogenesis (map00010) and for the closely related Pyruvate Metabolism (map00620) were also downregulated (**Table 3**). Pyruvate, a product of glycolysis, is a key component of the TCA cycle. Thus, the downregulation of these two pathways in the leaves could further indicate decreased energy processing in P-deficient leaf tissues. Collectively, the downregulation of these energy providing pathways in the transcriptome supports the idea that smaller P-deficient black cottonwoods are in a lower energy state.

P deficiency altered C allocation within black cottonwood seedlings, leading to greater reductions in the biomass of the woody tissues compared to the roots and the leaves of P-deficient black cottonwoods (**Figure 1B**). Differential regulation of C and energy metabolism as exhibited in both the transcriptome and the metabolome of the roots and the leaves of P-deficient black cottonwoods supported the observed phenotypes of increased root-to-shoot and leaf-to-shoot ratios of dry biomass (**Figure 1B**; **Tables 3**, **4**). Several putative mechanisms are highlighted in the transcriptomic and metabolomic data that may underlie this change in C allocation. First, in the leaf transcriptome, three genes encoding beta glucosidases were strongly downregulated (log_2_FC = –12 and –11, **Supplementary Table 3**). Among a range of functions, beta glucosidases can hydrolyze precursors to lignin (Dharmawardhana et al., 1995). In addition, in the root transcriptome, a gene encoding HXXXD-type acyl-transferase family protein (shikimate O-hydroxycinnamoyltransferase) was strongly downregulated (log_2_FC = –10, **Supplementary Table 3**). Shikimate O-hydroxycinnamoyltransferase is involved in the phenylpropanoid pathway, which is critical for the biosynthesis of lignin (Hoffmann et al., 2003, 2004). In the metabolome, the KEGG pathway for Starch and Sucrose Metabolism (map00500) was upregulated in leaf tissues (**Table 4**). Among its many functions, sucrose can also act as a signaling molecule to regulate C allocation during abiotic stress (Chiou and Bush, 1998). Therefore, the upregulation of sucrose metabolism in the leaves of P-deficient plants could be caused by a shift in C allocation to root growth for nutrient scavenging. Indeed, Shinde et al. (2017) noted large shifts in nonstructural carbohydrate profiles in *P. tremuloides* under P deficiency, with decreases in leaf concentrations and increases in root concentrations that may be associated with targeted root growth and exudation for P acquisition from the rhizosphere. Overall, in the transcriptome, 8 KEGG pathways assigned to the “energy” functional category were regulated in either direction across the leaves and the roots (**Tables 2**, **3**). However, the total number of DEGs and the number of regulated GO and KEGG pathways was higher in the leaf transcriptome than the root transcriptome (**Figure 2**; **Tables 1**, **2**). All of these suggest that P-deficient black cottonwoods adjust their C allocation, with the leaves eliciting a greater regulatory response than the roots, likely in order to prioritize resource acquisition instead of aboveground woody biomass.

Paralleling the sharp decrease in chlorophyll fluorescence in P-deficient plants, photosynthesis-related genes were downregulated in nutrient deficient black cottonwoods (**Figure 1D**; **Table 3**). Decreases in chlorophyll *F_v_/F_m_* are directly linked to decreases in the efficiency of photosystem II and indicate both the presence of photo-oxidative stress and declines in photosynthetic capacity (Kitajima and Butler, 1975; Krause and Weis, 1991; Guidi and Calatayud, 2014; Hernandez and Munne-Bosch, 2015; Guidi et al., 2019). In the leaf transcriptomes of P-deficient black cottonwoods, the KEGG pathway corresponding to Flavonoid Biosynthesis (map00941) was upregulated, potentially indicating plant response to light-induced oxidative stress during P-inhibited photosynthesis (**Table 2**). Moreover, Carbon Fixation in Photosynthetic Organisms (map00710) as well as Porphyrin and Chlorophyll Metabolism (map00860) were downregulated in the transcriptome (**Table 3**). The downregulation of these same KEGG pathways in roots most likely reflects a reduction in root C metabolism, such as glycolysis, resulting from limited photosynthesis in the leaves and restricted sucrose import.

The lesser decline (44% reduction) in leaf P concentration compared to roots (65% reduction) could be a result of a higher degree of membrane lipid remodeling in the roots or the capacity of roots to utilize/retranslocate inorganic P storage pools to a greater extent than P in leaves and supply that P to leaves to support photosynthesis (Haling et al., 2016). In times of severe P deficiency, plants can replace membrane phospholipids with non-P-containing lipids to free up P while maintaining membrane integrity via membrane lipid remodeling (Essigmann et al., 1998; Nakamura, 2013; Zhang et al., 2014b; Lan et al., 2015). Although several KEGG pathways related to membrane lipid remodeling were downregulated in both the leaves and roots, Sphingolipid Metabolism (map00600) was upregulated in both tissues (**Table 3**). Specifically, the roots had downregulated genes annotated to the KEGG pathways for Glycerophospholipid Metabolism (map00564) and Glycosphingolipid Biosynthesis Globo-Series (map00603), as well as the GO term Glycerolipid Biosynthetic Process (GO:0045017) (**Tables 1**, **2**). The leaves, on the other hand, only exhibited downregulation of the KEGG pathway for Glycerolipid Metabolism (map00561) (**Table 2**). By recovering P from membrane phospholipids in roots, P may have been reallocated to processes limiting photosynthesis in times of P deficiency, such as RuBP regeneration (Jacob and Lawlor, 1992; Hernandez and Munne-Bosch, 2015). From our data, it appears that P-deficient black cottonwoods use membrane lipid remodeling and retranslocation to a greater degree in their root tissues to help support whole-plant internal P homeostasis. Indeed, fatty acid metabolism genes encoding 3-ketoacyl-CoA synthase 6 and the AMP-dependent synthetase and ligase family protein were upregulated in the roots (Yang et al., 2018; Chevalier et al., 2019). These upregulated fatty acid metabolism genes are supported by the metabolomics data, which showed a pronounced shift in the relative proportion of phosphate ion in the roots, but not the leaves, which may reflect this remodeling and/or reduced incorporation of P into other metabolites perhaps limited by photosynthate delivery under P deficiency.

Out of the ten pathways differentially expressed in the metabolome, four were also differentially expressed in the transcriptome of the P-deficient black cottonwoods (**Tables 2**–**4**). These pathways were related to cellular respiration, amino acid biosynthesis, antioxidants, and starch metabolism (map00020; map00400; map00053; map00500; **Tables 2**–**4**). There are several possible explanations for the specific regulation of these pathways across the two -omes. The TCA pathway was upregulated in the leaf transcriptome and altered in the root metabolome, suggesting that the products of that pathway are being used to acclimate to low P status. The TCA cycle has been previously implicated in the production of organic acids in P-deficient tree roots (Chen et al., 2021). Coupled with the upregulation of the aluminum-activated malate transporter 1-related gene, linked to malate exudation and P scavenging, these changes suggest that black cottonwood seedlings likewise direct physiological function towards P scavenging from the rhizosphere under P deficiency (De Bang et al., 2021). In *P. tremuloides*, Desai et al. (2014) noted enhanced organic acid, including malate, exudation from roots of plants under P deficiency, supporting the upregulation of organic acid transporter genes as a coordinated response to P deficiency in black cottonwood as well. In addition, the regulation of the aromatic amino acid biosynthesis pathway across the -omes was likely related to the roles of phenylalanine and tyrosine in times of abiotic plant stress, where their accumulation protects plant tissues by the production of anthocyanins through the phenylpropanoid pathway (Christie et al., 1994; Gläßgen et al., 1998; Huang and Jander, 2017) (map00400; **Tables 2**, **4**). We have observed similar changes in secondary metabolite accumulation in *P. tremuloides* under P deficiency (Shinde et al., 2017). Although they were not the same pathway, two separate KEGG pathways involved in the production of the building blocks for P-rich nucleotides were differentially expressed across the root metabolome (map00230) and the transcriptome of both tissues (map00240), similar to the findings of previous studies (Zrenner et al., 2006) (**Tables 2**, **4**). The downregulation of these P-rich compounds could be a strategy used by black cottonwoods to conserve their limited supply of P. Overall, the areas of agreement between the transcriptome and the metabolome on processes like the biosynthesis of aromatic amino acids or nucleotides lend strong support to these pathways being important regulators of P deficiency response in black cottonwoods.

On the other hand, the direction of the regulation (upregulation vs. downregulation) of the other six pathways differentially expressed in both the transcriptome and the metabolome of the P-deficient black cottonwoods was inconsistent across the two -omes. For example, the pathway that regulates Starch and Sucrose Metabolism (map00500), a multi-functional pathway that controls the production and storage of carbohydrates and regulates signaling, was upregulated in the leaf metabolome but downregulated in the transcriptome of both tissues (**Tables 2**, **4**). We propose two mechanisms that could potentially explain the discrepancy between the responses of the Starch and Sucrose Metabolism (map00500) pathway and the remaining five pathways observed in the transcriptome and the metabolome of the P-deficient black cottonwoods. The first mechanism is related to the timeline across which the plants were exposed to the P deficiency stress. We observed end-point transcriptomes and metabolomes after a long-term treatment. By the harvest date, the metabolites of the Starch and Sucrose Metabolism (map00500) pathway were likely accumulated in the leaves. This accumulation could inhibit the synthesis pathway, resulting in a suppression of this pathway in the transcriptome. Alternatively, the pathway for Starch and Sucrose Metabolism (map00500) includes both the synthesis and the catabolism of the resultant metabolites. As such, the suppression of starch synthesis in P-deficient plants would lead to the downregulation of the Starch and Sucrose Metabolism (map00500) pathway in the transcriptome, while an accumulation of starch building blocks due to elevated starch catabolism in the P-deficient leaf tissues would appear as an upregulation of the Starch and Sucrose Metabolism (map00500) in the metabolome. Because starch catabolism can be used to provide energy in times of limited photosynthesis, we expected this process to be amplified in our P-deficient black cottonwoods (Thalmann and Santelia, 2017). However, there is also some variability in the regulation of starch content in response to different abiotic stressors, with some stresses leading to starch storage and others leading to an overall depletion of starch content (Thalmann and Santelia, 2017). Therefore, in addition to the transcriptomic and metabolomic changes to Starch and Sucrose Metabolism (map00500) observed in this study, more detailed explorations of specific starch and sucrose metabolic processes are needed to fully understand the roles of these molecules in black cottonwood P deficiency.

We found few differentially expressed proteins in black cottonwood seedlings and no significantly enriched GO terms in the black cottonwood proteome in response to P deficiency. This result was surprising given changes to both upstream (transcriptome) and downstream (metabolome) responses (Lan et al., 2015). Alignment of multi-omics is not always parsimonious (Lan et al., 2012), which could be due to the establishment of a new stress-induced homeostasis, multiple levels of gene regulation not captured here, or plant regulation of protein abundance being dependent on the intensity of P deficiency (Lan et al., 2015). Our conservative statistical approach and limited sample size may have also masked proteomic responses. We corrected our proteomics outputs in order to decrease the false discovery rate, which may have potentially excluded many pathways. Due to logistical concerns, we only conducted transcriptomic, metabolomic, and proteomic analysis on 5 plants per treatment group, which also limited our statistical power. Future studies should analyze plants that undergo different levels of P deficiency and durations to observe more dynamic aspects of the transcriptomic and metabolomic responses observed in this study.

### 4.1 Conclusions

Our results contribute to ongoing efforts to uncover the relationship between the phenotypic and molecular responses of black cottonwood leaf and root tissues to P deficiency. Considering the prevalence of P deficiency in soils, our results which showed that P-deficient black cottonwoods allocated less biomass to the woody tissues and regulated gene expression more strongly in the leaf tissues have strong implications for using black cottonwood as bioenergy feedstocks. We also showed that different -omes can show either harmonious or conflicting responses to P deficiency in black cottonwood, suggesting that observing a single -ome may conceal potentially important details of the P deficiency response in black cottonwood. Overall, this study demonstrates that future research into black cottonwood nutrient deficiency stress should use multi-omics approaches with additional focus on the tissue-specificity of plant response to resolve the mechanisms of interaction between tissues. Areas representing specific targets for tree selection for nutrient-limited environments include focus on the allocation of carbon flux to the rhizosphere via organic acid transporters to enhance P scavenging from soils. Future experiments should be expanded to include, for example, multiple P concentrations and multiple time points to resolve the distinction between -omic responses specific to P deficiency and responses that may be indicative of general stress.

## Data availability statement

The transcriptomics datasets generated for this study can be found in the Sequence Read Archive. The accession numbers for this dataset can be found in **Supplementary Materials 1**. The proteomics datasets generated for this study can be found in the ProteomeXchange under the project ID PXD046227.

## Author contributions

EK: Writing – original draft, Writing – review & editing. EB, RB, SC, EZ, Y-MK, PL & JC : Writing – review & editing.
